# Rapidly obtaining genome sequence of Severe Fever with Thrombocytopenia Syndrome virus directly from clinical serum specimen using long amplicon based nanopore sequencing workflow

**DOI:** 10.1371/journal.pone.0321218

**Published:** 2025-04-25

**Authors:** Tingting Tian, Yanhan Wen, Liping Gao, Tiezhu Liu, Xiaoxia Huang, Chuan Li, Shanshan Du, Hao Li, Meijun Guo, Jiandong Li, Shiwen Wang, Dexin Li, Aqian Li, Mifang Liang

**Affiliations:** 1 National Key Laboratory of Intelligent Tracking and Forecasting for Infectious Disease, National Health Commission Key Laboratory of Medical Virology and Viral Diseases, National Institute for Viral Disease Control and Prevention, Chinese Center for Disease Control and Prevention, Beijing, China; 2 China CDC-WIV Joint Research Center for Emerging Diseases and Biosafety, Center for Biosafety Mega-Science, Chinese Academy of Sciences, Wuhan, China; University of Pécs: Pecsi Tudomanyegyetem, HUNGARY

## Abstract

Severe Fever with Thrombocytopenia Syndrome (SFTS) is an emerging viral infectious disease discovered in 2009 with a high fatality rate and continuing to pose a public threat for many countries. Surveillance of genome sequence of its causative pathogen, Severe Fever with Thrombocytopenia Syndrome virus (SFTSV), could provide evidence for SFTS control, diagnosis method update, viral evolution dynamic and pathogenic mechanism research, etc. Here, we developed a workflow for rapidly obtaining the genome sequence of SFTSV directly from clinical samples to facilitate the viral genome sequence surveillance. Three pairs of primers targeting the terminal conserved regions of three segments were newly designed to more efficiently enrich nearly whole viral genome. Datasets comprised reads generated in different timeframes for four simulated samples with high to low serially diluted viral loads were subjected to analysis. For a simulated sample with a Ct value of 35 and sequenced for 10 minutes, the average coverage depth could reach over 700x, and the genome coverage could reach 98.69% after subtraction of the primer sequence, and the sequence identity with Sanger sequencing could reach over 99.91%. Two clinical serum specimens were used to validate the workflow and sequences were successfully obtained. A long amplicon based nanopore sequencing workflow was established, which could finish in 10 hours from serum specimen to genome sequence. This workflow has potential to provide essential information for SFTS control and support further pathogenesis research.

## Introduction

In 2009, an emerging infectious disease manifesting with fever, thrombocytopenia, leukocytopenia and a high case fatality rate was discovered in China, and named as Severe Fever with Thrombocytopenia Syndrome (SFTS) [1]. A novel bunyavirus initially named SFTS bunyavirus (SFTSV) was isolated and identified as the pathogen causing SFTS. As of Dec. 2023, more than 27 thousands of SFTS cases were reported in China with an annual fatality rate ranging from 2.7% to 12.7% [2]. After firstly found in China, SFTS was also reported in South Korea [3], Japan [4], Thailand [5] and United States [6]. In recent years, the incidence of SFTS increased, and the spatial distribution of SFTS has been observed to expand, making SFTSV an increasingly important regional or global potential threat to public health.

SFTSV, belongs to *Bunyaviricetes* class, *Hareavirales* order, *Phenuiviridae* family, *Bandavirus* genus *Bandavirus dabieense* species. Its genome contains three single-stranded negative-sense RNA segments (L, M and S segment), encoding nucleoprotein (NP), nonstructural protein (NSs), glycoprotein precursor and RNA dependent RNA polymerase (RDRP) [7]. In the whole genome with a size of 11 kb, mutation, recombination and reassortment all have been found [8,9]. It has been known that the genomic variations could reveal the evolutionary process and be associated with the disease severity [10]. Newly emerging variants had prompted the need to better understand genome features of SFTSV to update the diagnosis method, support further evolution process and pathogenesis research.

Sanger sequencing and next generation sequencing (NGS) are two widely used strategies to obtain genome sequence of virus now. While owing to the following benefits, like generating long reads, ability to real-time sequencing, portability as hand-size device and no need for corrosive chemicals like NaOH solution, nanopore-based sequencing is playing an increasingly important role in viral disease diagnosis and genome research. Those advantages make it suitable for field investigation of infectious disease outbreaks and monitoring of pathogen genomic variations. It has been used in sequencing for Ebola virus [11], SARS-CoV-2 [12], Lassa virus [13], Zika virus [14], monkeypox virus [15], and so on. Metagenomic sequencing is a widely used strategy to get the genome sequence of virus, while it maybe not suitable for specimen with low viral load. Here, we developed a novel long amplicon based workflow to enrich the SFTSV genome, together with nanopore sequencing technology, enabling rapid genome sequencing in the field.

## Methods

### Ethics statement

This study was approved by the Ethics Review Committee of the National Institute for Viral Disease Control and Prevention, Chinese Center for Disease Control and Prevention (IVDC2018–006).

### Simulated sample and clinical sample preparation

The SFTSV AH12 strain and two clinical serum specimens were provided by National Institute for Viral Disease Control and Prevention, Chinese Center for Disease Control and Prevention.

AH12 strain was isolated from serum and purified for three times by agarose plaque test on Vero cell line. The AH12 strain was serially diluted to 1/1,000, 1/10,000, 1/100,000 and 1/1,000,000 in healthy human serum to create four simulated samples with different viral loads, named with S1, S2, S3 and S4.

Two clinical serum specimens from patients with name C1, C2 were collected in 2014, and stored in a -80 °C freezer. To validate this long amplicon based sequencing workflow, these two specimens were taken out from the specimen library in October 2022. Authors did not have access to information that could identify individual participant after sample collection.

### Nucleic acid extraction and real-time PCR quantification

Viral RNA was extracted from 140 uL simulated samples or clinical serum specimens using QIAamp Viral RNA mini kit (QIAGEN, Hilden, Germany) as the manufacturer’s instruction. Real-time PCR was performed using AgPath-ID One-step RT-PCR Reagents (Applied Biosystems, Carlsbad, USA) as previously reported [16].

### Preparing long amplicons

First, three pairs of specific primers were designed (Table 1), each of which covered nearly the whole genome of L, M, S segment, respectively. Then, the virus genome was enriched using the specific primers and SuperScript IV UniPrime One-Step RT-PCR System (Invitrogen, Carlsbad, USA) in 3 reactions. A modified program were performed as follows: 56°C 30min, 98°C 2min, (98°C 10s, 60°C 10s, 72°C 4min)*40, 72°C 5min. An additional 65°C 30 minutes annealing process for RNA template and primers before the one-step RT-PCR was recommended. After purification with 1.8x Agencourt AMPure XP Beads (Beckman, California, USA), the S, M and L segment were mixed in equal amounts of 70 fmol each. If the amount of any segment was less than 70 fmol, add all the purified PCR product.

**Table 1:** The position and sequence of specific primers.

Segment	Primer name	Position^a^	Sequence
S	SFTSV-SF	19-39	AAGGAAAGACGCAAAGGAGTG
	SFTSV-SR	1722-1744	ACACAAAGACCCCCTTCATTTGG
M	SFTSV-MF	1-21	ACACAGAGACGGCCAACAATG
	SFTSV-MR	3356-3378	ACACAAAGACCGGCCAACACTTC
L	SFTSV-LF	1-19	ACACAGAGACGCCCAGATG
	SFTSV-LR	6344-6368	ACACAAAGACCGCCCAGATCTTAAG

^a.^NC_018137, NC_018138 and NC_018136 were used as reference sequences.

### Nanopore sequencing

After being phosphorylated the 5’ end and added an A-overhang to the 3’ end, each sample was ligated with a unique barcode using Native Barcode Expansion Kit (Oxford Nanopore Technology, Oxford, UK) and measured. Then, samples were pooled at an equal mole and were ligated with adapter using Ligation Sequencing Kit SQK-LSK109 (Oxford Nanopore Technology, Oxford, UK). Finally, the library was dropped into the flow cell R9.4.1 (Oxford Nanopore Technology, Oxford, UK) and sequencing started.

The raw data in the form of fast5 file was generated with sequencer running, including current change record, experiment time and other sequencing information. Guppy v6.4.2 (Oxford Nanopore Technology, Oxford, UK) was used to transform the electricity signal into base signal (base calling) with high accuracy (HAC) model, demultiplex barcodes, trim barcodes and adapters, filter low quality reads with Q score less than 9. All qualified reads were mapped to reference genome (NC_018136, NC_018137, NC_018138) using medaka v1.11.3 (Oxford Nanopore Technology, Oxford, UK) to get a draft sequence. Then, all qualified reads were mapped to the draft sequence using medaka v1.11.3 again to get the final consensus sequence. Cutadapt v4.8 was used to trim the amplification primer sequences from the terminal of consensus sequence.

### Sanger sequencing

A volume of 5uL one-step RT-PCR products were added to agarose gel to do the electrophoresis experiment. The gel cubes containing band at near 1.7kb, 3.3kb, 6.3kb position were cut and purified to get targeted DNA fragment. All samples were sequenced using Applied Biosystems 3730 xl Sequencer (Applied Biosystems, Carlsbad, USA). CAP3 [17] was used to assemble the genome sequence.

### Performance evaluation

Samtools v1.18 [18] and Bedtools v2.31.1 [19] were used to deal with the bam file generated by Medaka to get the number of reads mapped to the reference genome and coverage depth of each position of genome. Mosdepth v0.3.8 [20] was used to calculate the average coverage depth.

Mummer4 [21] was used to align the sequences acquired from nanopore sequencing and Sanger sequencing to get the identity and number of variants, indels. Pearson correlation test was used to explore the relationship. The R package tidyverse, ggplot2 and gggene were used to manipulate data and visualize.

## Results

### Establishing the long amplicon based nanopore sequencing workflow

**Fig 1:**
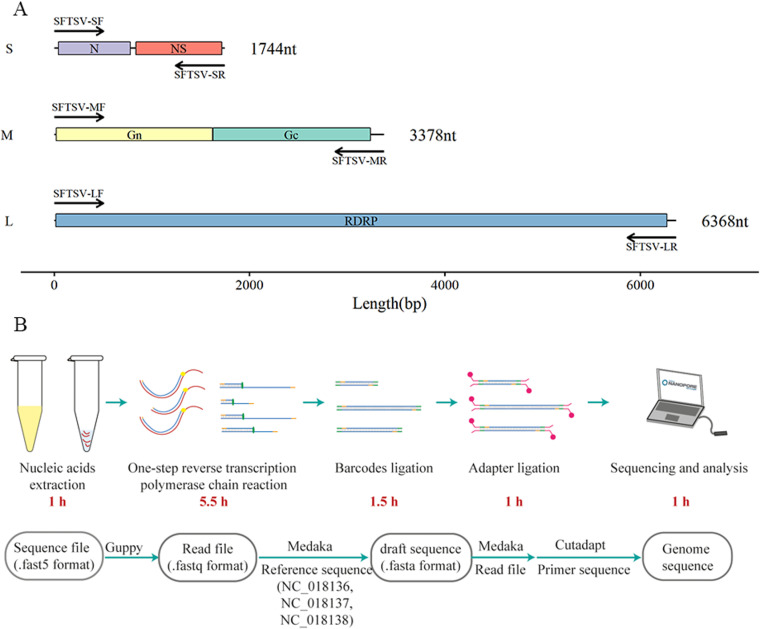
Establishing the long amplicon based nanopore sequencing workflow. A. The three pairs of primers’ location in the genome of SFTSV. B. The workflow of obtaining genome sequence of SFTSV from clinical serum specimen using nanopore sequencing technology in 10 hours. Main processes included nucleic acids extraction, genome sequence target enrichment, nanopore sequencing library preparation, real-time sequencing and analyzing.

To effectively enrich the genome, three pairs of specific primers covering nearly the whole genome of SFTSV were newly designed (Fig 1A). Then, a long-amplicon based workflow from the serum specimen to sequence was established (Fig 1B), which could be completed within 10 hours. Detailed procedures are illustrated in the method section, including 1 hour for nucleic acids extraction, 5.5 hours for genome sequence target enrichment with three pairs of newly designed primers, 2.5 hours for nanopore sequencing library preparation, such as barcode ligation, pooling samples and adapter ligation, and 1 hour for sequencing and data processing. A two-round mapping strategy using medaka was adopted. In the second round of mapping, the consensus sequence from the first round was taken as the reference genome.

### Summary of the simulated sample sequencing experiment

**Fig 2:**
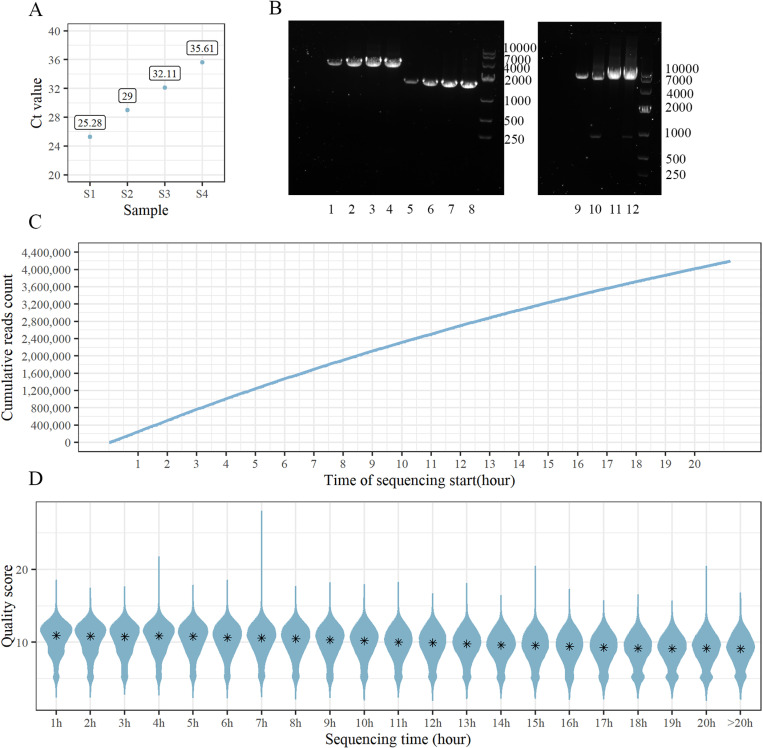
Summary of the simulated sample sequencing experiment. A. The quantitative result of four simulated sample ranges from 25.28 to 35.61. B. Clear and precise bands showed high efficiency of the newly designed primers. Lane 1, 2, 3, 4 were M segment for S4, S3, S2, S1, whose size was nearly 3.4 kb. Lane 5, 6, 7, 8 were S segment for S4, S3, S2, S1, whose size was nearly 1.7 kb. Lane 9, 10, 11, 12 were L segment for S4, S3, S2, S1, whose size was nearly 6.4 kb. C. The number of cumulative reads increased with the sequencing time. D. The density distribution of reads quality score generated in every hour. The asterisk indicated the median quality score of reads, which decreased with time.

RNA extracted from simulated samples S1-S4 containing gradient diluted AH12 strain in healthy human serum were quantified by SFTSV real-time PCR test. The Ct value ranged from 25.28 to 35.61 (Fig 2A). And then the samples were subjected to one-step RT-PCR for enrichment of viral genome. The new primers were shown to successfully amplify the target segment, indicating that the assay developed here has the capacity to obtain nearly complete L, M and S segments (Fig 2B).

The flow cell for S1-S4 initially contained 1378 pores at the start of the run. Over time, the number of reads increased rapidly (Fig 2C) and the median quality score of reads generated in each hour decreased (*P*<0.05, *r*=-0.99) (Fig 2D). Finally, a total of 4,190,330 reads were generated in 21 hour 12 minutes. Overall, the average sequencing speed was 415 bases per second. A proportion of 87.67% reads exhibited a quality score more than 7, and 67.99% reads had a quality score greater than 9. Within the first one hour, the number of reads had reached 245,046 with the corresponding median quality score of 10.94. It is worth noting that within only 10 minutes we obtained 30,437 reads with a median quality score of 11.14.

### The coverage depth for different sequencing time

**Fig 3:**
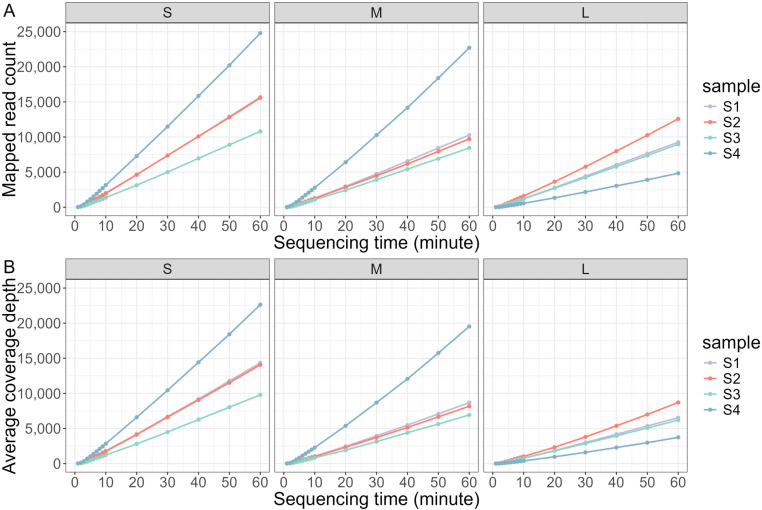
The mapped read count and average coverage depth for every segment in the first 1 hour. With the sequencing time passing, the mapped read count(A) and the average coverage depth(B) increased.

**Fig 4:**
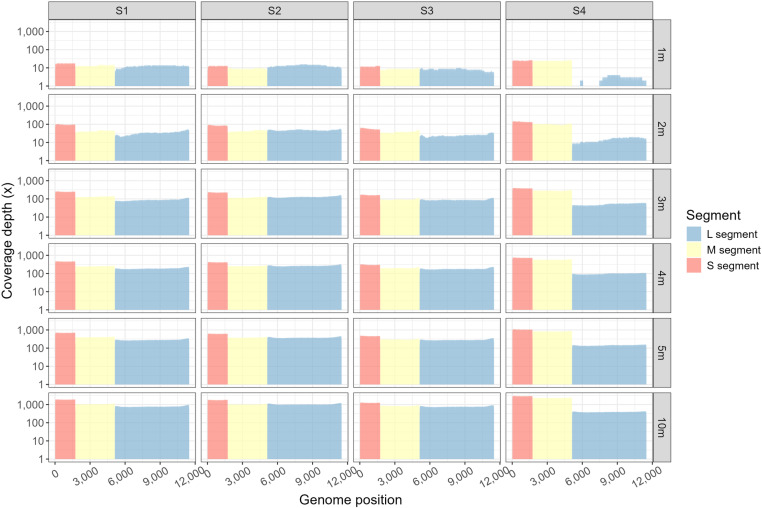
The coverage depth for simulated samples. An evenly distributed sequencing depth can be seen in each simulated sample’s whole genome. The figures in the first line showed the coverage depth for sequencing S1, S2, S3, S4 for 1minutes. The figures in the second, third, fourth, fifth, sixth line showed the coverage depth for sequencing S1, S2, S3, S4 for 2, 3, 4, 5, 10 minutes, respectively.

For each simulated sample, fifteen datasets containing reads which were generated during the first 1, 2, 3, 4, 5, 6, 7, 8, 9, 10, 20, 30, 40, 50, 60 minutes were produced and subsequently mapped to reference genome to generate a draft sequence. For a given sequencing time and segment, the Ct value showed no significant correlation with the number of mapped reads (*P*>0.05), or the average coverage depth (*P*>0.05), which indicated that specimen with low viral load could get similar mapped read count and coverage depth as those with high viral loads. With the sequencing time passing, the mapped read count for each segment increased (*r*> 0.99, *P*<0.01) (Fig 3A), so did average coverage depth (*r*> 0.99, *P*<0.01) (Fig 3B). After 4 minutes of sequencing, the average coverage depths for all simulated samples were over 60×. By 10 minutes of nanopore sequencing, all the four simulated samples showed over 1200× and 800× average coverage depth for S and M segment, respectively (S1 Table). Although sample S4, which contained the lowest viral load, showed a slightly lower average coverage depth (approximately 400×) for L segment, other three samples maintained coverage depths over 700×. Long amplicon based nanopore sequencing workflow provided an evenly distributed sequencing depth throughout the genome for all samples after 2 minutes of sequencing (Fig 4).

### The genome coverage for different sequencing time

Datasets of reads generated in the first 10, 20, 30, 40, 50, 60 minutes were mapped to the draft sequence to get consensus sequence. After subtracting the sequence of primers, the length of consensus sequence was 11,340 bp (1682 bp for S segment, 3334 bp for M segment and 6324 bp for L segment), accounting for 98.69% (11,340/11,490) of the reference sequence.

### The identity percent with Sanger sequencing

To validate the accuracy of this workflow, industry-standard Sanger sequencing was performed. The sequences obtained from nanopore sequencing datasets at 10, 20, 30, 40, 50 and 60 minutes were compared with those from Sanger sequencing using Mummer4 software, and the identity percent was calculated.

For a given simulated sample and segment, nanopore sequencing time showed no significant correlation with identity (*P*>0.05). Similar for a given sequencing time and segment, identity did not have a significant correlation with Ct value (*P*>0.05). For S4 sample, which had the lowest viral load, the sequence identity between 10 minutes of nanopore sequencing and Sanger sequencing reached at 100%, 100%, 99.91% for S, M and L segment, respectively. It was suggested that nanopore sequencing offered advantages in rapidly obtaining genome sequences of low viral load samples.

**Fig 5:**
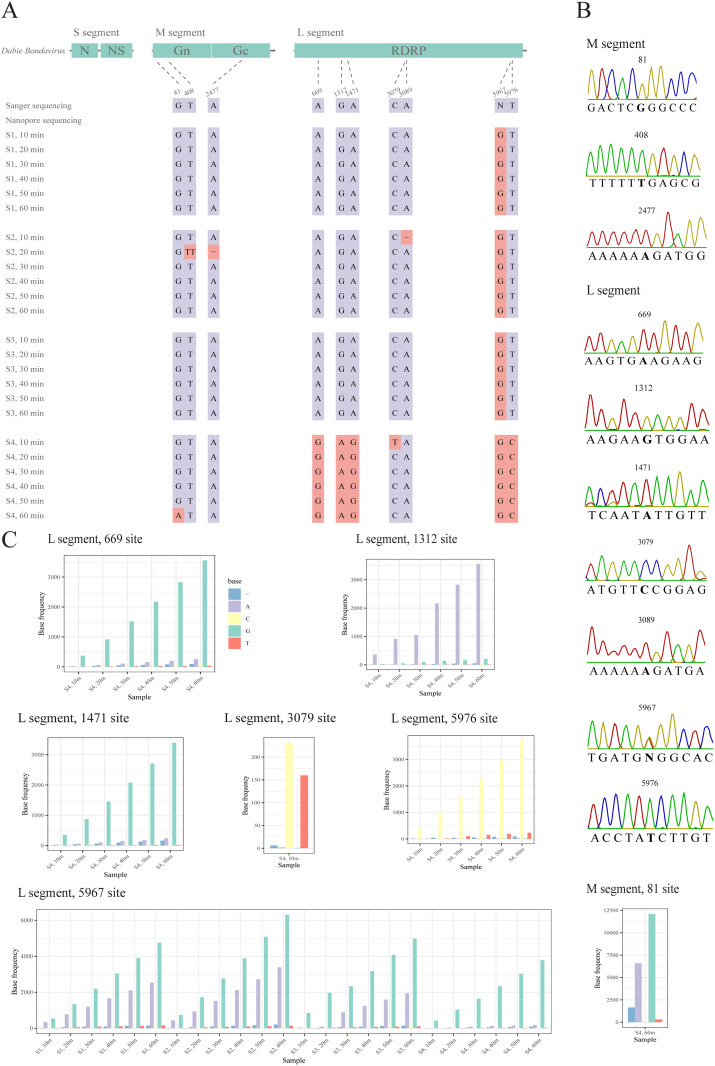
Distribution of unidentical sites from Sanger sequencing. A. A total of 10 unidentical sites between Sanger sequencing and nanopore sequencing distributed over the simulated sample whole genome. B. For M segment, 81, 408 and 2477 positions were in homopolymerize regions. For L segment, site 3089 was in the poly A region. C. Except homopolymer sites, all other unidentical sites’ second most base was identical with the Sanger sequencing.

Unidentical sites between sequences that were got from nanopore sequencing and Sanger sequencing were displayed in Fig 5. There was no unidentical sites in S segment. For M segment, 81, 408 and 2477 positions were unidentical sites. The sites 81 was surrounded by poly G. The sites 408, 2477 were in the poly T, poly A region, respectively (Fig 5B.). For L segment, unidentical sites were observed at positions 669, 1312, 1471, 3079, 3089, 5967 and 5976. The site 5967 was an AG heterozygous site, which was recognized as G in all simulated samples. The site 3089 which appeared only in S2 was in the poly A region. The unidentical site 669, 1312, 1471, 5976 only appeared in S4, and the second most frequent base was consistent with the Sanger sequencing results. The site 669, 1312, 5976 had the same bases with their neighborhood and the second most base was identical with the Sanger sequencing (Fig 5C). These findings indicated that an unidentical site frequently occurred in the region of homopolymers or near the homopolymers.

For the same specimen, no relationship was observed between time and identity (*P*>0.05). Similarly, no relationship was found between Ct value and sequence identity (*P*>0.05). However, the number of unidentical sites in the L segment S4 sample was higher compared other samples.

To improve the sequence identity, a second round of medaka was employed in the workflow. The identity of M segment obtained after the second round of medaka was significantly (*P*<0.05) higher than that from the first round, supporting the necessity of this additional step (S2 Table). The obtained sequence’s identity applied by SUP and HAC model showed no significantly different (*P*>0.05).

To further explore the limit of sequencing time, the sequences obtained from nanopore sequencing datasets at 1, 2, 3, 4, 5, 6, 7, 8, 9 minutes were compared with those from 10 minutes. After 2 minutes’ sequencing, the identity (S3 Table) was not significantly different with 10 minutes’ sequencing result (*P*>0.05).

### Validation using clinical serum specimens

To validate this workflow, two serum specimens, C1 and C2, provided from clinical confirmed patients was analyzed. The Ct value for C1 and C2 was 28.41 and 28.95, respectively. The flow cell for C1 and C2 initially had 1328 active pores at the start of the run. In the first 10 minutes, 58,193 reads were generated, of which 72.97% (42,465/58,193) had a quality score more than 9.

After ten minutes of sequencing, a total of 4,916 reads were mapped to C1 sample. The average coverage depth for S, M, L segment was 1810.38x, 1317.09x and 516.95x, respectively. A consensus sequence in total length of 10,339 bp (1682 bp for S segment, 3334 bp for M segment, 6323 bp for L segment) was generated. Comparing with Sanger sequencing, the identity for S, M and L segment reached 100%, 100% and 99.98%, respectively.

**Fig 6:**
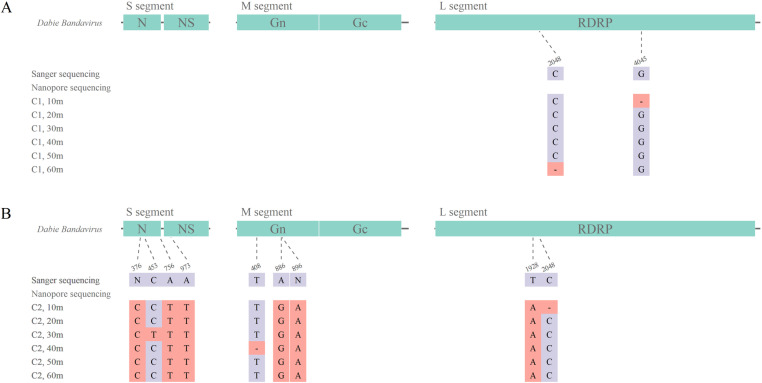
The unidentical sites distribution on the clinical serum specimen. A. For C1 sample, 2 unidentical sites were in the poly C and poly G region. B. For C2 sample, site 408 and 2048 were in the poly T and poly C region.

For the C2 sample, a total of 5,433 reads were mapped. The average coverage depth for S, M and L segment was 2489.32x, 1559.81x and 122.71x, respectively. A total length of 10,339 bp consensus sequence (1682 bp for S segment, 3334 bp for M segment, 6323 bp for L segment) was generated. Comparing with Sanger sequencing, the identity for S, M and L segment reached 99.82%, 99.94% and 99.97%, respectively. The unidentical sites were shown in Fig 6.

## Discussion

Surveillance of genomic sequence of SFTSV is an important work to reveal the virus evolution process, explore pathogenic mechanism, update diagnosis method, help control the SFTS outbreaks, etc. Metagenomic sequencing, PCR amplification sequencing and probe-based enrichment sequencing are three mainly strategies to get the viral whole genome sequence from clinical specimen [22]. Among them, PCR amplification strategy is suitable for specimen with known pathogens and it costs much lower than the others. Amplicon based nanopore sequencing of Ebola virus genomes provided a new, portable, real-time method during the 2014–2016 Ebola virus disease outbreak in West Africa, which suggested that PCR combined with nanopore sequencing technology is a promising tool for obtaining emerging and remerging viruses genome sequencing in poor conditions. For virus whose genome segment’s size falls within the range of a single PCR extension length and has highly conserved terminal sequences, long amplicon sequencing strategy has been widely applied, like Influenza A virus [23], *Orthohantavirus* species [24] and Dengue virus [25]. Considering that SFTSV also has suitable genome size and highly conserved terminal sequence, we tried to establish a long amplicon based nanopore sequencing workflow to access the genome sequence of SFTSV.

In this study, we newly designed 3 pairs of primers with high amplification efficiency and chose a real time nanopore platform capable of sequencing long nucleic acid fragments, established long amplicon based nanopore sequencing workflow. Then we sequenced 4 simulated samples and analyzed 15 datasets containing reads generated in the first 1, 2, 3, 4, 5, 6, 7, 8, 9, 10, 20, 30, 40, 50, 60 minutes since the sequencing test started. Finally, we validated the whole workflow with clinical serum specimens. In our experiment, we could get the genome sequence of SFTSV in 10 hours.

Different with multiplex PCR strategy using 115 pairs of primers [26], we chose long-amplicon method and designed only three pairs of primers. There are two advantages in long-amplicon workflow. First, the sequences obtained through long-amplicon are derived entirely from the viral genome. Each segment was amplified by only one pair of primers. In conjunction with single-molecule nanopore sequencing technology, a single read could cover nearly the whole segment. Thus our result could minimize the risk of introducing artificially designed sites than multiplex PCR strategy and reduced horizontal coverage in some regions due to the primer-template mismatch annealing [27]. Second, long-amplicon workflow’s primers are robust theoretically. A widely recognized fact is that there are conserved regions at the terminals of three segments of the SFTSV genome, where primers were all located. They are more robust to be applied for wide range of SFTSV specimens, despite of the presence of numerous variant sites and recombination or reassortment events, which sometimes lead to PCR failure [28]. It could be found that using multiplex PCR workflow, 99.20% for the S segment, 99.47% for the M segment, 99.43% for the L segment were got while using long-amplicon workflow, 98.69% for the whole genome was got. And the consensus sequence covered nearly the whole open reading frame, which was enough for further analysis. While it is crucial that the RNA templates remain intact to get enough long amplicons. To meet with this requirement, a good storage of specimen is necessary. Since that, this workflow is efficient to get SFTSV genome sequence, and cannot replace qPCR test.

To test the workflow’s performance in low viral load specimen, we diluted AH12 virus strain with healthy people serum to prepare a series 10-fold dilutions of simulated samples with different viral loads and sequenced them in one flow cell. Low viral load specimen with a Ct value of 35 could get similar mapped read count, coverage depth, identity with Sanger sequencing, as high viral load specimen. All above demonstrated that the workflow reported in this study was applicable for specimens with low viral load that was difficult to analyze efficiently using metagenomic sequencing strategy.

Since nanopore sequencing could produce reads at a rate of approximately 400 bp/s from the onset of the experiment, it is feasible to obtain genome sequences within a relatively short timeframe. We investigated the optimal and minimum time required to achieve sufficient genome sequencing coverage. When the sequencing time reached 10 minutes, all segments of simulated samples could have an average coverage depth of more than 300x. Rowena’s research showed that sensitivity and precision of variant detection decline sharp below ~50x coverage depth, and show slight improvement above 60x coverage depth for nanopore sequencing [29]. Therefore, it could be considered that a sequencing time of 10 minutes was sufficient to meet the requirement of enough coverage depth.

For simulated samples, sequences’ identity exceeded 99.91% with Sanger sequencing technology. No significant correlation was observed between identity and sequencing time. So, in only 10 minutes we could get the consensus sequence. An additional round of medaka could improve the identity, while it should be noted that it may be only beneficial for the use of R9.4 flow cell data. Unidentical sites seemed frequently located in the regions with homopolymers or near the homopolymers. Beate’s research on influenza A virus also found the same phenomenon [30]. As a single-molecular sequencing method, R9.4 nanopore flow cell accuracy is 85%-94% [31], not as much as Sanger sequencing and the second generation sequencing. But as the new nanopore protein, motor enzyme design and algorithms come out, the accuracy of reads will improve. New R10.4.1 flow cell and updated basecalling software have already released, the accuracy is greatly improved.

Finally, we validated the workflow using clinical serum specimens. After 10 minutes of sequencing, we got over 99.8% identity consensus sequence compared with Sanger sequencing, suggesting this workflow’s application in serum. These results demonstrated that it is an excellent sequencing strategy for field investigation and could save precious time for disease control. However, there are also some limits. An increased number of clinical specimens with varying range of viral loads should be advocated to be used for the workflow validation. Besides, exploring alternative library strategies, like using rapid library chemicals, faster reverse transcriptase and DNA polymerase could further reduce the turnaround time consuming.

## Conclusion

As the causative agent of SFTS, SFTSV attracts much attention, and the surveillance of genomic sequence has become an essential task. In this study, a long amplicon based nanopore sequencing workflow that enabled the genome sequencing from the serum finished in 10 hours was established. The whole workflow was evaluated and determined that it was appropriate for low viral load with Ct value as low as 35. Genome sequence obtained using this workflow demonstrated an identity exceeding 99.91% compared to those generated by Sanger sequencing. This workflow showed the potential to facilitate genome sequencing and thus conserving valuable time for SFTS control.

## Supporting information

S1 Table The average coverage depth for simulated samples. (PDF)

S2 Table Comparison the identity of consensus sequence obtained from the first round of medaka and second round of medaka. (PDF)

S3 Table The limit of sequencing time. (PDF)
