# Supplementary material for: Rapidly obtaining genome sequence of Severe Fever with Thrombocytopenia Syndrome virus directly from clinical serum specimen using long amplicon based nanopore sequencing workflow
Source: PLoS One. 2025 Apr 25;20(4):e0321218. doi: 10.1371/journal.pone.0321218 (PMC12027057; doi:10.1371/journal.pone.0321218)
Supplement: S1 Table — (PDF) [file pone.0321218.s001.pdf]

S1 Table. The average coverage depth for simulated samples

Sample	Minute	S	M	L
S1	10	1825.30	1042.88	772.94
S1	20	4180.72	2448.65	1853.62
S1	30	6692.58	3955.93	3009.79
S1	40	9191.54	5480.90	4171.62
S1	50	11778.93	7081.27	5357.30
S1	60	14346.01	8693.15	6528.86
S2	10	1730.39	1012.81	1022.70
S2	20	4129.03	2330.78	2330.57
S2	30	6612.46	3709.95	3802.04
S2	40	9055.01	5146.24	5372.28
S2	50	11524.15	6659.42	6995.03
S2	60	14067.76	8165.49	8703.89
S3	10	1215.40	830.61	762.42
S3	20	2803.81	1915.22	1807.19
S3	30	4488.53	3130.23	2860.79
S3	40	6270.66	4397.61	3947.97
S3	50	8033.09	5632.23	5067.00
S3	60	9777.7	6923.11	6188.56
S4	10	2832.88	2279.19	388.47
S4	20	6588.37	5379.04	968.71
S4	30	10442.43	8688.85	1600.95
S4	40	14422.59	12059.42	2289.04
S4	50	18422.55	15738.69	2973.72
S4	60	22634.49	19534.02	3736.54
