# Supplementary material for: Rapidly obtaining genome sequence of Severe Fever with Thrombocytopenia Syndrome virus directly from clinical serum specimen using long amplicon based nanopore sequencing workflow
Source: PLoS One. 2025 Apr 25;20(4):e0321218. doi: 10.1371/journal.pone.0321218 (PMC12027057; doi:10.1371/journal.pone.0321218)
Supplement: S2 Table — (PDF) [file pone.0321218.s002.pdf]

S2 Table. Comparison the identity of consensus sequence obtained from the first round of medaka and second round of medaka

Sample	Minute	S		M		L	
		Identity 1st round (%)	Identity 2nd round (%)	Identity 1st round (%)	Identity 2nd round (%)	Identity 1st round (%)	Identity 2nd round (%)
S1	10	100	100	99.97	100	99.98	99.98
S1	20	100	100	99.91	100	99.98	99.98
S1	30	100	100	99.97	100	99.98	99.98
S1	40	99.94	100	99.94	100	99.98	99.98
S1	50	99.94	100	99.94	100	99.98	99.98
S1	60	100	100	99.97	100	99.98	99.98
S2	10	100	100	99.94	100	99.98	99.97
S2	20	100	100	99.94	99.94	99.98	99.98
S2	30	100	100	99.97	100	99.98	99.98
S2	40	100	100	99.97	100	99.98	99.98
S2	50	100	100	99.97	100	99.98	99.98
S2	60	100	100	99.97	100	99.98	99.98
S3	10	100	100	99.94	100	99.98	99.98
S3	20	100	100	99.91	100	99.97	99.98
S3	30	100	100	99.97	100	99.98	99.98
S3	40	100	100	99.94	100	99.98	99.98
S3	50	100	100	99.91	100	99.98	99.98
S3	60	100	100	99.97	100	99.98	99.98
S4	10	100	100	99.97	100	99.91	99.91
S4	20	100	100	99.97	100	99.91	99.92
S4	30	100	100	99.94	100	99.92	99.92
S4	40	100	100	99.94	100	99.92	99.92
S4	50	100	100	99.94	100	99.92	99.92
S4	60	99.94	100	99.97	99.97	99.92	99.92
