# Supplementary material for: Rapidly obtaining genome sequence of Severe Fever with Thrombocytopenia Syndrome virus directly from clinical serum specimen using long amplicon based nanopore sequencing workflow
Source: PLoS One. 2025 Apr 25;20(4):e0321218. doi: 10.1371/journal.pone.0321218 (PMC12027057; doi:10.1371/journal.pone.0321218)
Supplement: S3 Table — (PDF) [file pone.0321218.s003.pdf]

S3 Table. The limit of sequencing time

Sample	Minute	S		M		L	
		Coverage depth	Identity (%)	Coverage depth	Identity (%)	Coverage depth	Identity (%)
S1	1	16.94	99.94	13.14	99.94	12.30	99.97
S1	2	93.57	99.94	41.92	99.97	32.99	99.98
S1	3	242.06	100	130.00	100	86.55	99.98
S1	4	446.36	100	248.68	99.97	187.47	99.97
S1	5	677.26	100	396.75	100	279.92	99.97
S1	6	887.68	100	514.19	100	382.23	99.98
S1	7	1103.19	100	635.77	100	472.45	99.98
S1	8	1325.46	100	761.72	100	572.49	99.98
S1	9	1565.62	100	906.47	100	681.41	99.98
S2	1	12.19	100	8.93	99.97	13.08	99.94
S2	2	83.97	100	42.56	99.97	47.46	99.97
S2	3	220.39	100	118.49	99.97	127.93	99.97
S2	4	404.04	100	253.81	99.97	264.11	99.97
S2	5	597.73	100	376.66	100	374.62	99.97
S2	6	830.35	100	498.59	99.97	495.94	99.97
S2	7	1048.69	100	620.44	99.97	630.01	99.97
S2	8	1239.95	100	749.81	99.97	760.03	99.97
S2	9	1482.27	100	880.73	99.97	884.15	99.98
S3	1	11.47	100	8.56	99.91	8.02	99.92
S3	2	55.03	100	37.10	100	24.24	99.97
S3	3	158.87	100	92.08	100	87.56	99.98
S3	4	294.74	100	196.58	100	178.04	99.98
S3	5	449.08	100	299.35	100	280.23	99.98
S3	6	620.65	100	390.69	100	363.34	99.98
S3	7	774.49	100	500.29	100	476.01	99.98
S3	8	901.77	100	623.31	100	581.14	99.97
S3	9	1051.62	100	720.34	100	664.85	99.97
S4	1	24.46	100	24.27	99.97	2.3	97.18
S4	2	133.91	100	99.74	100	14.41	99.91
S4	3	373.06	100	282.2	100	50.56	99.91
S4	4	712.28	100	568.62	100	96.2	99.91

S4	5	1024.26	100	822.82	100	141.47	99.91
S4	6	1375.77	100	1117.72	100	190.16	99.91
S4	7	1730.96	100	1414.98	100	231.98	99.91
S4	8	2088.92	100	1695.77	100	288.81	99.91
S4	9	2444.97	100	1986.72	100	339.29	99.91
